# Stochastic model of IP_3_-induced Ca^2+^ spiking of HEK293 cells

**DOI:** 10.1371/journal.pcbi.1013322

**Published:** 2025-08-22

**Authors:** Caterina Azzoni, Rene Jüttner, Anje Sporbert, Michael Gotthardt, H. Llewelyn Roderick, Martin Falcke

**Affiliations:** 1 Max Delbrück Center for Molecular Medicine (MDC), Berlin, Germany; 2 Faculty of Life Sciences, Humboldt-Universität zu Berlin, Berlin, Germany; 3 DZHK (German Centre for Cardiovascular Research), Partner Site Berlin, Berlin, Germany; 4 Charité-Universitätsmedizin, Berlin, Germany; 5 Department of Cardiovascular Sciences, Experimental Cardiology, KU Leuven, Belgium; 6 Department of Physics, Humboldt-Universität zu Berlin, Berlin, Germany; University of Kiel Faculty of Medicine: Christian-Albrechts-Universitat zu Kiel Medizinische Fakultat, GERMANY

## Abstract

Mathematical theory that accounts for the stochastic character of spike sequences of IP_3_-induced Ca^2+^ signalling calculates the probability distributions of the features of the [Ca^2+^]_*i*_ time course, their moments and correlations. Including slow feedback from [Ca^2+^]_*i*_ to components of the pathway poses a challenge to stochastic modelling. Here, we present a stochastic model that takes this feedback into account, allows for a non-linear dependency of the open probability of the Inositol 1,4,5-trisphosphate receptor channel (IP_3_R) on the feedback variable and the inclusion of more than one feedback with different relaxation time scales. We use this novel modelling approach to describe the effect of ER depletion by non-linear rate expressions for Ca^2+^-induced Ca^2+^ release (CICR) and the measured non-linear IP_3_-dependency of the open probability as part of the dynamic feedback. Our theory can calculate spike amplitude distributions, correlation coefficients (C_*c*_) of interspike intervals (ISIs) and amplitudes, simulate ISI distributions and calculate their moments. We apply it to experiments with HEK293 cells. We find very good agreement between theoretical ISI distributions and their moments with experimental results. Many measured C_*c*_s show positive values in accordance with the ideas formulated by our theory. Surprisingly, most ISI-amplitude correlations are weak despite the decay of negative feedback during the ISI, which affects spike probability. We even find negative values of C_*c*_s, which indicate feedback that decreases the open probability of IP_3_R with increasing ISI. The components of the pathway causing this anticorrelation have not yet been identified. Our data suggest that they involve components that are subject to cell variability.

## Author summary

Ca^2+^ is a versatile second messenger. Ca^2+^ signals show stochastic spike timing and large cell variability despite their signal transmission function. Spike train properties, which are not subject to cell variability and thus define the system, are related to noise. Hence, the first step in theoretical comprehension must be a stochastic theory. This study reports on progress in developing that theory and on experimental results. The application to Ca^2+^ signals in HEK293 cells meets basic expectations and, in part, explains the surprising results on ISI-amplitude correlations. The measured anticorrelation requires us to expand our ideas on feedback during spiking and the corresponding stochastic theory. Once we understand the role of fluctuations, we may also derive deterministic approximations for simulating averages.

## Introduction

**Fig 1:**
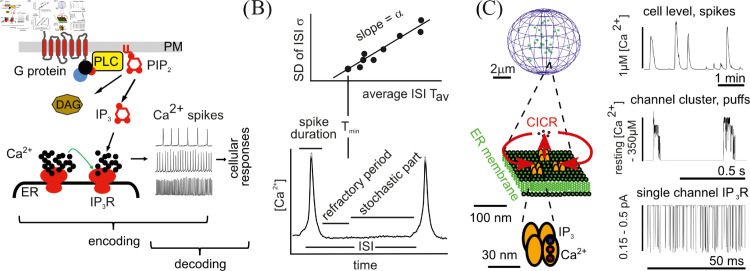
(A) Inositol 1,4,5-trisphosphate (IP_3_) pathway of Ca^2+^ signaling. Binding of an agonist to one of a large family of G-protein coupled receptors (GPCR) activates phospholipase C (PLC), which produces IP_3_ from phosphatidylinositol-4,5-bisphosphate (PIP_2_). IP_3_ sensitises IP_3_ receptor channels (IP_3_R) in the endoplasmic reticulum (ER) membrane to binding of Ca^2+^, such that Ca^2+^ released from the ER through open channels increases the open probability of closed channels by Ca^2+^-induced Ca^2+^-release (CICR). PLC activates additional feedback to the IP_3_R open probability via diacylglycerol (DAG). (B) The interspike interval (ISI) comprises the spike duration, the refractory period, and the stochastic part *t*_*sto*_. The sum of spike duration and refractory period is T${\;\;}_{\text{min}}$. It is a parameter of the theory and constant throughout a spike train. The sum of T${\;\;}_{\text{min}}$ and a realisation of *t*_*sto*_ (T${\;\;}_{\text{min}}$ + *t*_*sto*_) is a realisation of an ISI. The average ISI is denoted T${\;\;}_{\text{av}}$ and the average of *t*_*sto*_ is T${\;\;}_{\text{sto}}$. T${\;\;}_{\text{av}}$=T${\;\;}_{\text{min}}$+T${\;\;}_{\text{sto}}$ holds. T${\;\;}_{\text{av}}$ and the standard deviation (SD) of ISI *σ* are linearly related with slope *α*. (C) IP_3_-induced Ca^2+^ signalling is hierarchical with channels (comprising a tetramer of IP_3_R proteins) on the lowest structural level, channel clusters as intermediate structure and cluster arrays on the cell level. Each structural level has its typical time scale and signal (see [1–5] for local currents and concentrations). The state of the IP_3_R is determined by IP_3_ and Ca^2+^ binding to the IP_3_R tetramer. Channel clusters are the elementary units of cellular dynamics. Their release events are Ca^2+^ puffs generated by CICR. CICR entails excitability. Clusters cooperate by CICR in generating cellular Ca^2+^ spikes as global events. This study considers the dynamics of spike generation by clusters under global feedback that acts on all clusters.

IP_3_-induced Ca^2+^ signalling transmits information that arrives in the form of an agonist concentration at the cell plasma membrane to intracellular targets through changes in cytosolic [Ca^2+^]_*i*_ [6–11]. We introduce the pathway and some terms and definitions in Fig 1. Intracellular Ca^2+^ signals may be local/subcellular (puffs), sequences of [Ca^2+^]_*i*_ spikes (see Fig 1), or a long-lasting increase in [Ca^2+^]_*i*_ (overstimulation) [8,12]. The variability of the Ca^2+^ signals and of the average interspike interval (T${\;\;}_{\text{av}}$) among cells stimulated with the same concentration of agonist is large [9,13]. Furthermore, the interpuff interval (IPI) and the interspike interval (ISI) are random [9,13,14]. This randomness and variability in the response to an identical stimulation (identical initial signal) in an information-transmitting system raises several basic questions, most notably how such a system can transmit information at all. This question has found a partial answer: The average frequency of a spike sequence is the inverse of the average ISI. Agonist concentration steps induce changes in the average of the stochastic part of ISI in all cells by the same factor [9]. This can be likened to a melody which is a sequence of sound frequency ratios. Since a sequence of agonist concentration steps elicits the same sequence of frequency ratios in all cells, all cells play the same melody. However, due to the large variability of the average ISI between cells, each cell plays at its own pitch [9]. We still do not know how the cell-to-cell variability of the pitch can be reconciled with the signal transmission function of the system.

Concerning biophysical modelling, the conceptual questions to be addressed are: 1) what should be modelled given the lack of a unique response, 2) how can models be parameterised given variability and randomness, 3) which general rules can models reveal despite variability and randomness. The cumulant relation between the standard deviation (SD) of ISI and the average ISI introduced one general rule, which states that its slope *α* is conserved within cell variability and is specific to a cell type and agonist [9,13,15–17]. The concentration-response relationship of the average ISI (mentioned above) revealed that the average ISI depends exponentially on the concentration of the agonist. The agonist sensitivity *γ* is the same for all individual cells stimulated with the same agonist. Cell variability affects only the exponential prefactor [9]. Both conserved properties relate to stochastic aspects of Ca^2+^ spiking. Although obvious for *α*, it also applies to *γ*, as stimulation controls the stochastic part of the ISI [9]. Several theoretical studies on stochastic aspects of Ca^2+^ signalling have been published to address the questions related to this system [17–31]. Here, we report progress in developing a theory that focuses on the calculation of ISI and amplitude statistics.

The [Ca^2+^]_*i*_ signal can be induced either by Ca^2+^ entry from the extracellular space through plasma membrane channels, or by Ca^2+^ release from intracellular storage compartments, primarily located in the endoplasmic reticulum (ER). In the following, we will focus on IP_3_-induced Ca^2+^ release from ER, which is the predominant Ca^2+^ release mechanism in many cell types (see Fig 1) [32].

The released Ca^2+^ is removed from the cytosol primarily by sequestration into the ER by sarco-endoplasmic reticulum Ca^2+^ ATPases (SERCAs) and to a lesser extent by extrusion by plasma membrane Ca^2+^ ATPases out of the cell.

IP_3_Rs are spatially organised into clusters with a variable number of channels from 1 to about 15 [33–37]. By including single channels in our cluster definition, we anticipate that the formulated theory will also be applicable to channel populations that do not cluster, as suggested by Lock et al. [37]. The clusters are scattered on the ER membrane with distances reported of 1 to 7 μm [38–42]. CICR and Ca^2+^ diffusion then couple the state dynamics of the channels and clusters. The coupling between channels in a cluster is much stronger than the coupling between adjacent clusters [1].

The structural hierarchy of IP_3_R arrangement from single channels to clusters and cellular cluster arrays is also reflected by the dynamic responses of the intracellular Ca^2+^ concentration, as revealed by fluorescence microscopy and simulations [20,38,43–45]. Random openings of single IP_3_Rs (blips) may trigger collective openings of IP_3_Rs within a cluster (puffs), while Ca^2+^ diffusing from a puff site can then activate neighbouring clusters, eventually leading to a global, i.e., cell-wide, Ca^2+^ spike [20,42,44–46]. Recent studies suggest that clusters generating puffs are preferentially located close to the plasma membrane and single IP_3_Rs dominate in the bulk of cells [37,47–49]. The timing of blips, puffs and spikes is random [9,13–17,22,25,26,45,50–52].

The typical spiking time scale is the average T${\;\;}_{\text{av}}$ of ISIs. We learn about processes that generate this time scale by considering the different structural levels. The local dynamics of Ca^2+^ signalling is the dynamics of isolated clusters. Their average interpuff intervals are 1-2 orders of magnitude shorter than T${\;\;}_{\text{av}}$ [53]. Interestingly, long sequences of puffs from isolated single puff sites do not exhibit slow modulations of the interpuff interval or puff amplitude on the T${\;\;}_{\text{av}}$ time scale [53]. However, interacting puff sites can form spikes; thus, the T${\;\;}_{\text{av}}$ time scale is an emerging property of cell-level dynamics. Once global, these dynamics elicit the negative feedback that terminates spikes. The balance between [IP_3_] and the recovery from the negative feedback which terminates spikes determines the length of the stochastic part *t*_*sto*_ of ISI. Upon maximal [IP_3_], a global elevation of [Ca^2+^]_*i*_ is induced that does not oscillate until the system overcomes its refractoriness.

Stimulation of cells by agonist binding to plasma membrane G protein-coupled receptors (GPCRs) (see Fig 1) activates pathways beyond IP_3_ production with receptor-specific feedback to IP_3_ levels and Ca^2+^ release [10,32,54–58]. These pathways also affect the negative feedback mechanisms that terminate release spikes. Recovery from this global negative feedback causes the slow increase in open probability during *t*_*sto*_. It may also cause an absolute refractory period immediately after the spike preceding *t*_*sto*_ (see Fig 1B) [9]. The negative feedback that determines the time scale of ISIs is different from the feedback that contributes to interpuff intervals (IPIs) and requires global release events.

In our experimental system, binding of carbachol (CCh) to muscarinic GPCRs leads to their activation and stimulation of downstream pathways involving PLC*β* isoforms and Protein Kinase C (PKC) [59,60]. PLC catalyses the hydrolysis of phosphatidylinositol 4,5-bisphosphate (PIP_2_) into IP_3_ and diacylglycerol (DAG) (Fig 1) [59,60]. Several studies report that PKC exerts negative feedback to IP_3_-induced Ca^2+^ release [61–64]. PKC activation requires binding of Ca^2+^ and DAG [64]: IP_3_ produced by PLC starts Ca^2+^ release in the cytosol, facilitating PKC translocation to the plasma membrane, where it can interact with DAG [64]. PKC phosphorylates and inhibits PLC*β*, thereby reducing IP_3_ production and exerting its negative feedback on Ca^2+^ release [59,64]. Corrêa-Velloso et al. report an increase in average ISI upon acute inhibition of PKC with ADP-stimulated hepatocytes, while observing no effect of PKC inhibition on average ISI in UTP-stimulated hepatocytes [65]. The authors also describe more complex PKC-mediated feedback affecting spike width.

Our theory exploits the hierarchical structure of Ca^2+^ signalling from single IP_3_Rs to clusters and cluster arrays at the cell level. We perceive spike generation as beginning with the opening of a first cluster. We define a cluster as open, if 1 or more of its channels are open. That first opening sequentially recruits more clusters by CICR until almost all available channel clusters are open, forming a global spike. This stochastic process progresses while the cell recovers from the negative feedback that terminated the previous spike. It can be mapped to a random walk with time-dependent transition probabilities on a linear state scheme indexed by the number of open clusters. The ISI distribution is the first passage time distribution from all clusters closed to almost all clusters open shifted by T${\;\;}_{\text{min}}$. The moments of this distribution can be determined analytically from the solution of a system of difference equations in Laplace space [66]. A proof-of-principle study demonstrated that this theoretical approach is capable of reproducing the basic general properties of Ca^2+^ spiking [67]. However, the theory was limited to the linear dependence of the transition probabilities on the inhibitory variable *I* that mediates the termination of the spikes by negative feedback.

We generalise the theory to non-linear dependencies on *I* in this study. This allows for much more realistic rate expressions and offers rules on how to turn biological ideas into stochastic theory. We illustrate this with a newly derived expression for the contribution of CICR to the transition rates and by implementing the dependence of the single-cluster puff rate on [IP_3_] measured by Dickinson et al. [35] into our theory. We introduce the calculation of spike amplitude distributions and the joint probability of ISI and spike amplitude. We parameterise our theory with data from HEK293 cells on the SD-T${\;\;}_{\text{av}}$ relation, amplitude variability, and ISI-amplitude correlation. Powell et al. suggested that ISIs of HEK293 cells obey a Γ-distribution [14]. We show that a Γ-distribution very well approximates our theoretical results.

## Results

### Mathematical model

**Fig 2:**
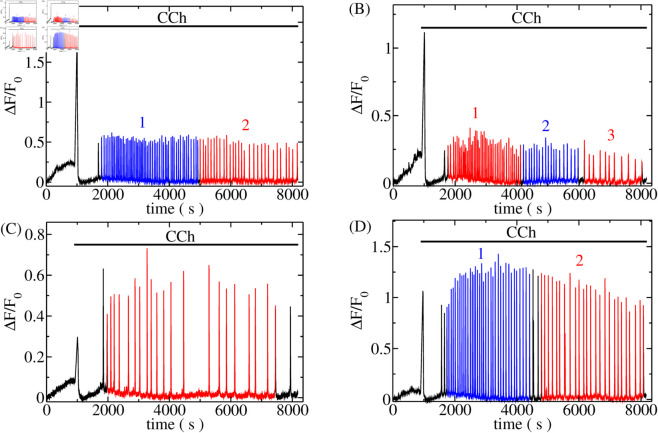
Example recordings of [Ca^2+^]_*i*_ spike trains of HEK293 cells, obtained as described in Sect Materials and methods. Panels A-C are spike trains of three different cells from the same experiment, illustrating cell variability. Panel D displays a cell spike train from a separate experiment with the same conditions as in A-C. All cells were stimulated with 15 μM carbachol (CCh) starting at 900 s. The onset of stimulation triggered an immediate [Ca^2+^]_*i*_ spike, the characteristics of which may depend on the initial Ca^2+^ store state. Colored segments exhibit approximately stationary spiking (see text and Sect D in the S1 Text) and were analysed individually. T${\;\;}_{\text{av}}$ value for A, segment 2 is 111.5 s; B, segment 1 is 54.5 s and segment 3 is 187.5 s; Panel D, segment 1 is 76.2 s and segment 2 is 134.2 s. See also Table 1 for information on other segments.

Global dynamics of intracellular [Ca^2+^]_*i*_ arise from the collective behaviour of individual channels, spatially arranged in clusters, that cooperate via CICR to generate spikes, as shown in the experimental records in Fig 2.

**Fig 3:**
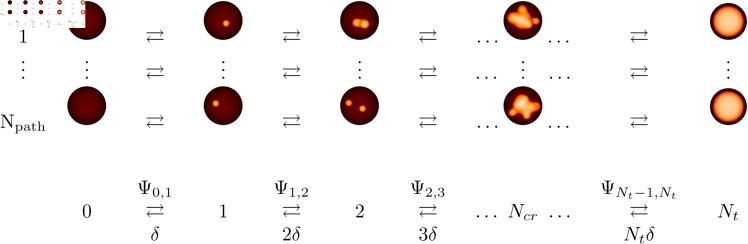
Schematic of cluster array dynamics. (Top) Open clusters are visualised as small orange spheres. A spike occurs when (almost) all *N*_*t*_ clusters are open. There are N${\;\;}_{\text{path}}$ paths of cluster openings and closing from 0 to *N*_*t*_ open clusters. (Bottom) Averaging over all paths leads to a state scheme indexed by the number of open clusters. See text for more explanations.

Our stochastic model tracks the state of the cluster array over time, describing its state by the number of open clusters (see Fig 3). The probability that the cluster array has *k* open clusters is denoted by *P*_*k*_. The dynamics of these state probabilities are given by the Master Equation

$$\frac{dP_{k}}{dt}=\Psi_{k-1,k}P_{k-1}+(k+1)\delta P_{k+1}-\left(\Psi_{k,k+1}+k\delta \right)P_{k},$$ (1)

with $k=0,\ldots ,N_{t}$, $\Psi_{-1,0}=\Psi_{N_{t},N_{t}+1}=0$ and $P_{N_{t}+1}=0$. Open clusters close with rate *δ*. Clusters open with the rate $\Psi_{k,k+1}$ (Fig 3), which depends on [IP_3_] *i*_*p*_ and [Ca^2+^] *c*. [IP_3_] is part of dynamic feedback and varies with time *t*. The same applies to [Ca^2+^] which additionally is affected by the number of open clusters *k*:

$$\Psi_{k,k+1}\left(i_{p},c,t\right)=g\left(i_{p}(t)\right)\cdot (N_{t}-k)\cdot r_{n}(c(k,t)).$$ (2)

*N*_*t*_ is the total number of clusters in the cell. The factor $g\left(i_{p}\right)$ describes the dependency of the single cluster puff rate on [IP_3_]. *N*_*t*_−*k* is the number of closed clusters, and *r*_*n*_(*c*) is the rate factor resulting from CICR. The subscript *n* denotes the number of Ca^2+^ ions required to bind to the IP_3_R to be in a state with high open probability.

The time dependence of $\Psi_{k,k+1}$ arises from the recovery of the cell from the negative feedback that terminated the previous spike with rate *λ*. It aggravates the solving of Eq 1 substantially. Fortunately, the Laplace transform ${\tilde{P}}_{k}(s)$ contains most of the information we are interested in. The Laplace transform of Eq 1 is a system of difference equations in Laplace space that can be solved analytically [66,67]. For the analytic solution to be applicable, we had to restrict the transition probabilities to linear dependencies on $e^{-\lambda t}$, which imposed constraints on the modelling of feedback. Here, we generalise the solution to arbitrary polynomial dependencies of the $\Psi_{k,k+1}$ on $e^{-\lambda t}$. The details are given in S1 Text, Sect A. The dependencies of the transition probabilities on $e^{-j\lambda t},j=1,\ldots ,N_{p}$ turn the system of first-order difference equations into a system of *N*_*p*_*th* order difference equations. We can reduce it to a system of first-order difference equations by the introduction of new variables $\tilde{P}(s{)}_{jN_{t}+k}(s)=\tilde{P}(s{)}_{(j-1)N_{t}+k}(s\;+\;\lambda )$, $j=1,\ldots ,N_{p}\;-\;1$, and we can solve this system of first-order difference equations by the solution given in [66,67] and Eqs A.8-A.13 in S1 Text. The moments of the ISI distribution are determined by Eqs A.16, A.17. The amplitude distribution is determined by Eqs A.18, A.19. We also simulated trajectories and compared their statistics to the analytical calculations.

The Taylor series illustrates that any function can be approximated by a polynomial. Hence, we can deal with any dependency of the transition probabilities on $e^{-\lambda t}$ now, which we use in this study to introduce more realistic feedback and two concomitant feedback. The Taylor series is usually not the best approximating polynomial for a given polynomial degree *N*_*p*_, since its convergence is guaranteed for $N_{p}\to \infty$ only. We will see below that excellent approximations can be found with a different choice of polynomial coefficients.

#### The relation between the single cluster puff rate and [IP_3_].

Dickinson et al. measured a linear relationship between puff frequency and the number of channels in a cluster [35]. This strongly suggests that channels within a closed cluster behave independently and identically to a very good approximation, and thus the cluster opening probability is proportional to the single channel opening probability.

**Fig 4:**
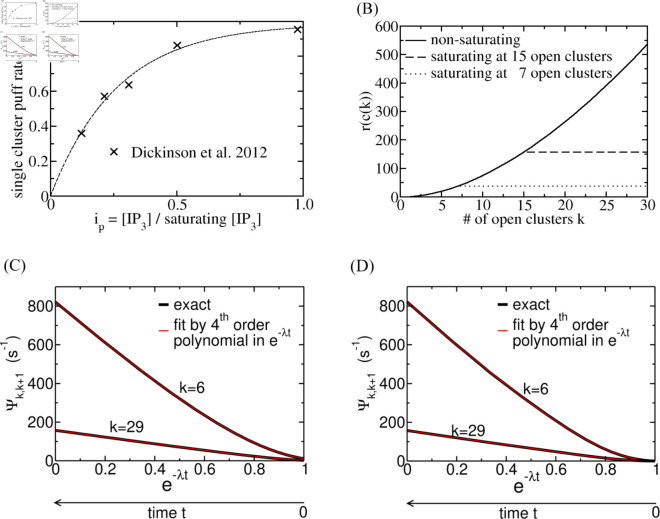
(A) Relation between the single cluster puff rate and [IP_3_]. Data points from Dickinson et al. [35] are marked by ×. They show a non-vanishing puff rate without uncaging of IP_3_, which might indicate a resting [IP_3_]. We shifted the data by this resting concentration. The line is Eq 3. As a remark, $c_{0}(1\;-\;e^{-c_{1}i_{p}})$ with appropriate values for *c*_0_ and *c*_1_ provides also excellent fits [67] but is not suitable for our analytical calculations. (B) The rate factor describing CICR Eq 10 and Eq 13 fully recovered (t=∞). (C) $\Psi_{k,k+1}$ (Eq 2) in dependency on $e^{-\lambda t}$ with constant [IP_3_] (*i*_*p*_ = 1) and recovering CICR (Eq 13). (D) $\Psi_{k,k+1}$ (Eq 2) in dependency on $e^{-\lambda t}$ with recovering *i*_*p*_ (Eq 4) and recovering CICR (Eq 13). (C, D) The fits by fourth-order polynomials for each *k* are indistinguishable from the exact relation. (B-D) Δ=0.95, *K*_*p*_ = 18, *s*_*p*_ = 2, *g*_0_ = 1 s^−1^, *N*_*t*_ = 30.

We now specify the factors on the rhs of Eq 2. The relationship between the single cluster puff rate and a scaled [IP_3_] has been measured by Dickinson et al. [35]. [IP_3_] has been controlled intracellularly by photo-liberation of IP_3_ from an inactive caged precursor in these experiments, and is thus known except for an unknown scaling factor. Consequently, we also use a scaled form *i*_*p*_ of [IP_3_], which is scaled with the concentration that saturates the single cluster puff rate. The puff rate measured by Dickinson et al. in SHSY-5Y neuroblastoma cells can be well approximated by

$$g\left(i_{p}\right)=g_{0}\left(4.816i_{p}-9.413i_{p}^{2}+8.379i_{p}^{3}-2.782i_{p}^{4}\right)$$ (3)

as shown in Fig 4A. It saturates at $g=0.937{\text{s}}^{-1}$ as in SHSY-5Y cells [35] with these coefficients of the polynomial and $g_{0}=1\;{\text{s}}^{-1}$. The saturation value might be up to 5 times larger in HEK293 cells than in SHSY-5Y cells [53]. [IP_3_] is controlled by stimulation with agonist concentration [*A*]. It might also be controlled by the negative feedback that ends the spike, making [IP_3_] a dynamic variable. Therefore, we describe *i*_*p*_ during the recovery from negative feedback like

$$i_{p}=i_{p}^{max}\left([A]\right)\left(1-e^{-\lambda t}\right),\;0\leq i_{p}^{max}\left([A]\right)\leq 1.$$ (4)

Time t = 0 is the end of the absolute refractory period of the previous spike in this equation. At this moment *i*_*p*_ = 0 and then recovers to an asymptotic value $i_{p}^{max}$ set by stimulation with [*A*].

A spike occurs at the time *t*_*sto*_ after the end of the absolute refractory period of the previous spike. Upon onset of the spike, IP_3_-targeting negative feedback decreases [IP_3_] during the spike and we describe it as follows:

$$i_{p}=i_{p}^{max}\left([A]\right)re^{-\lambda_{s}t}.$$ (5)

The second factor *r* describes the state of recovery of *i*_*p*_ at the end of the ISI preceding the spike. The cell has recovered to the degree

$$r=1-e^{-\lambda t_{sto}}$$ (6)

during the ISI preceding the spike. We call *r* the recovery variable. The last factor is the exponential decrease of *i*_*p*_ with the rate $\lambda_{s}$ since the onset of the spike at *t* = 0. This negative feedback to [IP_3_] contributes to the termination of the spike.

#### The rate factor describing CICR.

CICR increases the rate of cluster opening, and we derive a corresponding expression for the rate *r*_*n*_ in this section. We focus on the dependency of *r*_*n*_(*c*(*k*,*t*)) on *c* and *k* and neglect all factors that can finally be subsumed into a common factor of $\Psi_{k,k+1}$ like *g*_0_ in Eq 3.

We consider the stationary fraction of open channels dependent on [Ca^2+^] to define ideas about how [Ca^2+^] affects the channel opening rate. That fraction increases like [Ca^2+^]${\;\;}^{\text{n}}$ before reaching a maximum according to a variety of studies [68–73], with reported values for *n* from 1.0 to 4.0 (1.0–2.7 [71], 2.7 [68], 1.6 our fit to data from [72], up to 4.0 [70]). That suggests that IP_3_R has at least 3 Ca^2+^ ions bound in the state with a high open probability.

Assuming mass action kinetics of Ca^2+^ binding, we can write down a Master Equation for the probabilities *P*_*b*_(*i*) that the (tetrameric) IP_3_R has *i*= 0, 1, 2 Ca^2+^ ions bound. We use this Master Equation to calculate the average first passage time (FPT) of the bare receptor to the state with 3 Ca^2+^ ions bound. A standard method of calculating FPT distributions starts with solving the Master Equation with the condition *P*_*b*_(3) = 0 [74]:

$$\frac{dP_{b}(0)}{dt}=k^{-}P_{b}(1)-4{\bar{k}}^{+}P_{b}(0)\;\frac{dP_{b}(1)}{dt}=4{\bar{k}}^{+}P_{b}(0)+2k^{-}P_{b}(2)-(k^{-}+3{\bar{k}}^{+})P_{b}(1)\;\frac{dP_{b}(2)}{dt}=3{\bar{k}}^{+}P_{b}(1)-2(k^{-}+{\bar{k}}^{+})P_{b}(2)$$ (7)

The binding rate constant of Ca^2+^ ions is *k*^+^, and thus ${\bar{k}}^{+}=k^{+}$[Ca^2+^]. The dissociation rate is *k*^−^. The Laplace transform of this system of differential equations provides expressions for the Laplace transforms ${\tilde{P}}_{b}(i)$ of *P*_*b*_(*i*). The average FPT can then be determined from the derivative of 2${\bar{k}}^{+}{\tilde{P}}_{b}(2)$ as described in S1 Text, Eq C.2. We take the inverse of the average FPT as the rate of reaching the state with 3 Ca^2+^ ions bound:

$$r_{3}(c)=\frac{12k^{+}[{\text{Ca}}^{2+}{]}_{\text{r}}c^{3}}{13c^{2}+5K_{p}c+K_{p}^{2}},\;c=\frac{\left[{\text{Ca}}^{2+}\right]}{[{\text{Ca}}^{2+}{]}_{\text{r}}},\;K_{p}=\frac{k^{-}}{k^{+}[{\text{Ca}}^{2+}{]}_{\text{r}}}.$$ (8)

[Ca^2+^]_*r*_ is the resting [Ca^2+^]_*i*_. *K*_*p*_ is the ratio of the dissociation constant of the receptor Ca^2+^ binding site $\frac{k^{-}}{k^{+}}$ to [Ca^2+^]_*r*_. If 4 Ca^2+^ ions need to bind to reach the state with high open probability, we obtain:

$$r_{4}(c)=\frac{12k^{+}[{\text{Ca}}^{2+}{]}_{\text{r}}c^{4}}{25c^{3}+18K_{p}c^{2}+13K_{p}^{2}c+3K_{p}^{3}}.$$ (9)

Another factor of *r*_*n*_ comes from the picture of spike generation as wave nucleation [46,75–78]. We describe it in a very naive approach. The closed clusters near the expanding wave experience high [Ca^2+^] and dominate cluster opening. Their number increases as the surface of the volume is engulfed by the wave. The number of open clusters *k* determines that volume. Neglecting the factors subsumed into *g*_0_ we reach the expression:

$$r_{3}(c)=\frac{c^{3}{\left(1+k\right)}^{\frac{2}{3}}}{c^{2}+\frac{5}{13}K_{p}c+\frac{1}{13}K_{p}^{2}}.$$ (10)

$$r_{4}(c)=\frac{c^{4}{\left(1+k\right)}^{\frac{2}{3}}}{c^{3}+\frac{18}{25}K_{p}c^{2}+\frac{13}{25}K_{p}^{2}c+\frac{3}{25}K_{p}^{3}}.$$ (11)

We choose a simple linear relation between scaled [Ca^2+^] *c* and the number of open clusters by assuming quasi-stationary profiles of the cytosolic concentration. They reach their shape in the cytosol quickly upon opening or closing of clusters, but the dynamics of slow variables determining the cytosolic profiles, e.g. the ER filling state, slowly change the cytosolic concentration even with a constant configuration of open clusters [1,2,79].

The model [Ca^2+^] concentration variable *c* is the concentration in units of the resting concentration (Eq 8). It is an increasing and saturating function of the number of open clusters [1,2,79,80]. We set *c* = 1 + *kS*_*p*_(*t*). *S*_*p*_(*t*) quantifies how much a single open cluster increases *c* (in units of [Ca^2+^]_*r*_). If many but not all clusters are open, [Ca^2+^] is high at most closed clusters. The opening of more clusters will not increase [Ca^2+^] further for most closed clusters, since they are most likely not proximally localised to those that are opening. Hence, we describe saturation by limiting *c* to values reached at the number *k*_*s*_ of open clusters

$$c(k,t)=\left\{ \begin{array}{ccc} 1+kS_{p}(t) & , & k\leq k_{s}\\ 1+k_{s}S_{p}(t) & , & k\geq k_{s} \end{array}\right..$$ (12)

The upper bound *k*_*s*_ applies also to the factor $(1+k){\;\;}^{\frac{2}{3}}$ in Eqs 10, 11 and thus in the end fixes an upper bound to *r*_*n*_(*c*) (Fig 4).

*S*_*p*_(*t*) picks up the slow dynamics. We exemplify our ideas with ER depletion. The ER is (partially) depleted at the end of a spike, the release currents are reduced, and thus *S*_*p*_ is decreased. Furthermore, the luminal Ca^2+^ controls the IP_3_R gating [81]. We describe depletion by a factor 1−Δ, 0≤Δ≤1. The degree of depletion may be minor, causing little decrease in release rates and *S*_*p*_ (Δ≪1) or may cause a substantial decrease of *S*_*p*_ (Δ≈1). The ER slowly refills after the spike, i.e. the depletion factor approaches 1: $S_{p}(t)=s_{p}\left(1-\Delta e^{-\lambda t}\right)$. That leads to an expression for *c* during recovery from depletion like

$$c(k,t)=1+ks_{p}\left(1-\Delta e^{-\lambda t}\right).$$ (13)

The ER depletes during a spike, and thus we get

$$c(k,t)=1+ks_{p}\left(1-\Delta \left(1-re^{-\lambda_{s}t}\right)\right).$$ (14)

with t = 0 being the time of onset of the spike.

The time dependent *c* (Eq 13 or 14) enters *r*_*n*_(*c*) (Eq 10 or 11) and thus causes a dependency of the $\Psi_{k,k+1}$ (Eq 2) on $e^{-\lambda t}$: $\Psi_{k,k+1}\left(i_{p},c\left(k,e^{-\lambda t}\right),k\right)$. That dependency is shown in Fig 4C. It can be very well approximated by a fourth-order polynomial in $e^{-\lambda t}$.

#### Combining negative feedback to [IP_3_] and ER depletion.

Since each spike partially depletes the ER, this negative feedback is always present. Therefore, negative feedback to [IP_3_] always occurs in combination with ER depletion, and the $\Psi_{k,k+1}$ depend on $e^{-\lambda t}$ via *c* (Eqs 10, 11 and 13, 14) and *i*_*p*_ (Eq 3 and Eqs 4, 5): $\Psi_{k,k+1}\left(i_{p}\left(e^{-\lambda t}\right),c\left(k,e^{-\lambda t}\right),k\right)$. This complex dependency on $e^{-\lambda t}$ can also be well approximated by a fourth-order polynomial, as Fig 4D shows. The reasons for the high quality of these fits are that $e^{-\lambda t}$ only varies between 0 and 1, that the functions we deal with are smooth, and that we fit up to the saturating argument value, but not far into the saturated range.

In summary, we can find polynomial expressions capturing the time dependency of feedback for all the cases we considered, and thus we can broadly apply our analytic theory. This allows for a much larger variety of models than before [67]. We used the combined feedback throughout this study.

### The ISI distribution and its moments

**Fig 5:**
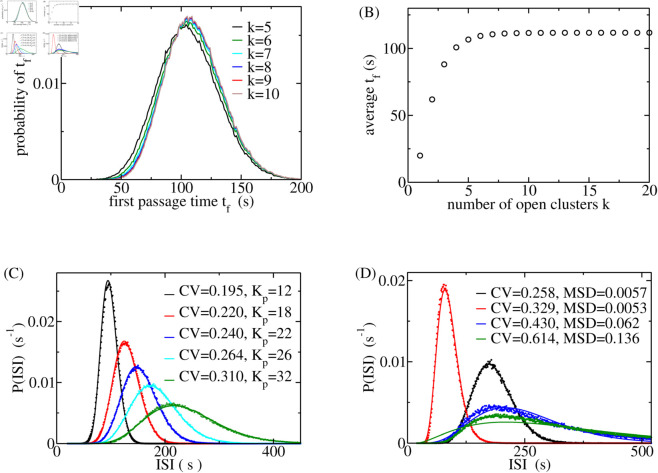
(A) The distributions of the FPT *t*_*f*_ from 0 to k open clusters. They are very similar for k = 6-10. (B) The average of the FPT *t*_*f*_ from 0 to k open clusters. The value of *t*_*f*_ at k = 7 (110.5 s) deviates by about 1% from the values wit k≥15 (111.6 s). Based on that criterion, we will use *k*_*sp*_ = 7 in our calculations of spike characteristics. (C) ISI distributions with varying Ca^2+^ dissociation constant *K*_*p*_ of the IP_3_R. CV is the coefficient of variation of the stochastic part of the ISI. The T${\;\;}_{\text{av}}$ values with increasing *K*_*p*_ are 77 s, 110 s, 135 s, 164 s and 217 s. (D) ISI distributions can be very well approximated by Γ-distributions if the CV is sufficiently small. We find an excellent or good approximation for CV ≤ 0.43, but not for CV = 0.614. MSD is the mean root of the squared deviation relative to the maximum of the Gamma distribution. (A, B, D) Δ=0.95, *N*_*t*_ = 30, *s*_*p*_ = 2, *k*_*s*_ = 15, *K*_*p*_ = 18, *g*_0_ = 1.0 s^−1^, $i_{p}^{max}$ = 0.1, *λ* = 0.01 s^−1^, (D) parameter values different from the common set: black *g*_0_ = 0.6 s^−1^, red *g*_0_ = 0.2 s^−1^, $i_{p}^{max}$ = 0.75, *λ* = 0.02 s^−1^, blue *g*_0_ = 0.3 s^−1^, *λ* = 0.02 s^−1^, green *K*_*p*_ = 32, *g*_0_ = 0.6 s^−1^, *λ* = 0.02 s^−1^.

The first step in determining the ISI distribution is to define what a spike is. Some cells exhibit release events larger than puffs, but still much smaller than spikes: the number of open clusters here is too small, and CICR has not yet reached the strength necessary to convert those events into global spikes. If the number of open clusters is large enough, CICR opens clusters in a fast sequence during the rising phase of the spike. We call a release event a spike if it is of size *k*_*sp*_, which is large enough to cause this fast rise. Since the upstroke is fast, the distributions of FPTs *t*_*f*_ to numbers of open clusters *k* larger than *k*_*sp*_ are very similar. Thus, we can determine *k*_*sp*_ as the smallest *k* in a sequence of *k*s with similar *t*_*f*_-distributions. We consider as a spike all release events with at least *k*_*sp*_ open clusters. The results in Fig 5A and 5B suggest *k*_*sp*_ = 7 to meet the definition. The distribution of *t*_*sto*_ is the distribution of FPTs from 0 to *k*_*sp*_ open clusters with this definition of a spike.

We show several examples of simulated ISI distributions with a varying Ca^2+^ dissociation constant of IP_3_R in Fig 5C. The larger the dissociation constant *K*_*p*_ the less likely it is that the receptor has 3 Ca^2+^ ions bound at a given [Ca^2+^]_*i*_, and the larger [Ca^2+^]_*i*_ transients are required to generate a spike. It takes larger opening probability $\Phi_{k,k+1}$ to generate these transients, which occurs later in the recovery process than the one required with small *K*_*p*_. Thus, the average ISI T${\;\;}_{\text{av}}$ increases with increasing *K*_*p*_. The Ca^2+^ sensitivity is regulated by ATP [71,82–85]. The IP_3_R-subtype 1 is about 3 times more sensitive in elevated [ATP] than in low [ATP], and the subtype 3 is about 10 times more sensitive. Our results suggest that a lack of ATP and a consequential decrease in the Ca^2+^ sensitivity increase the average ISI. This is in agreement with experiments by Betzenhauser et al. in DT40 cells investigating the properties of IP_3_R II [85]. They observed an increase in ISI and a decrease in spike amplitude upon mutating the relevant ATP-binding site on IP_3_R. Thus, cell-to-cell differences of [ATP] may be another parameter causing cell variability of the average ISI T${\;\;}_{\text{av}}$ besides parameters including number of clusters, specific geometry of the cluster array, density of plasma membrane receptors, SERCA density and more (see [67,86] for detailed discussions).

Powell et al. found that the experimentally determined ISIs of HEK293 cells obey Γ-distributions [14]. Therefore, we compare our simulated distributions with Γ-distributions with the same average and SD (Fig 5D) and find very good agreement.

We can relate the experimental results to the theoretical ISI distributions via moments, cumulants, their relationships and the coefficient of variation CV (CV = SD/average). The CV of the stochastic part of the ISI is well approximated by the slope of the cumulant relation between the average of the ISI T${\;\;}_{\text{av}}$ and the ISI SD *σ*

$$\sigma =\alpha \left({\text{T}}_{\text{av}}-{\text{T}}_{\text{min}}\right).$$ (15)

It is of particular interest, as we found that it is conserved despite the large variability in T${\;\;}_{\text{av}}$ and *σ* between cells under identical conditions and also in a variety of experimental situations [9,13]. That robustness is important since the smaller the value of *α*, the larger the information content transmitted by a spike sequence measured either as Kullback entropy with a Poisson distribution as reference [87,88] or mutual information between stimulating agonist concentration and T${\;\;}_{\text{av}}$ [89]. The Γ-distributions describing the HEK293 ISI statistics are two-parameter distributions with a shape and a rate parameter. The shape parameter is equal to CV^−2^ and is consequently as conserved as *α* and the same for all individual HEK293 cells in many different experimental situations [9,13].

There are two experimental observations related to *α* - its value for ISI in the range of T${\;\;}_{\text{av}}$$\approx \lambda^{-1}$, and the robustness of this value against cell variability. Both require some reflection. Recovery from negative feedback during the first passage process of spike generation is the reason why *α* has a value smaller than 1.0. The absence of recovery or very fast recovery, that is, λ≫T${\;\;}_{\text{av}}$^−1^, entails *α* = 1.0 [74,87,88]. From these considerations, it is clear that the rate of recovery from negative feedback *λ* sets the slope *α* of the cumulant relation Eq 15 [67,90].

If T${\;\;}_{\text{av}}$ is very large, i.e. T${\;\;}_{\text{av}}$$\;\gg \;\lambda^{-1}$, recovery is fast compared to T${\;\;}_{\text{av}}$ and *α* has values close to 1. Consequently, the T${\;\;}_{\text{av}}$ range through which we measure a typical value of *α* is another characteristic of the pathway. That defines the robustness requirements. The value of *α* should be approximately constant throughout the range of observed average ISI, and experiments suggest that this range is much larger than the smallest ISI [9,13].

**Fig 6:**
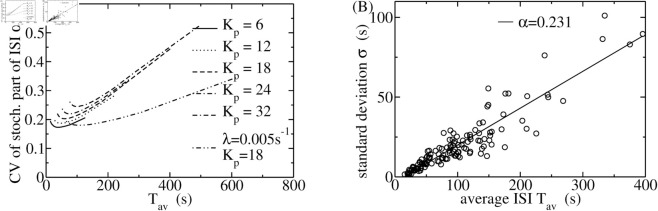
(A) The relationship between the CV of the stochastic part of the ISI *α* and T${\;\;}_{\text{av}}$ for a variety of *K*_*p*_- values. The range of T${\;\;}_{\text{av}}$-values of the data is caused by varying $i_{p}^{max}$ from 0.025 to 1.0. *α* stays within the measured range of HEK cells with *λ* = 0.005 s^−1^. Parameter values Δ = 0.95, *N*_*t*_ = 30, *s*_*p*_ = 2, *k*_*s*_ = 15, *g*_0_ = 1.0 s^−1^, *λ* = 0.01 s^−1^ if not indicated otherwise. Eq 10 has been used for CICR. (B) The cumulant relation Eq 15 was measured with HEK293 cells. Each data point represents results from one spike train. The slope *α* of the cumulant relation approximates the CV of the stochastic part of the ISI. The full line is a fit of a linear function to all data points with the indicated *α*-value. Fitting linear functions to the ranges 0 ≤ T${\;\;}_{\text{av}}$ ≤ 70 s, 70 s ≤ T${\;\;}_{\text{av}}$ ≤ 160 s and 160 s ≤ T${\;\;}_{\text{av}}$ ≤ 400 s provides slopes of 0.262. 0.219 and 0.280, i.e. we find a similar non-monotonic behaviour as in theory.

We are now in a position to assess our theoretical results and relate them to experimental observations. We show the results of calculations for the CV of the stochastic part for a range of *K*_*p*_ values for average ISI up to 400 s (Fig 6A). CV is moderately affected by *K*_*p*_. It shows a much stronger dependency on T${\;\;}_{\text{av}}$ with *λ* = 0.01 s^−1^ than with *λ* = 0.005 s^−1^. Therefore, we used *λ* = 0.005 s^−1^ in fits of our HEK293 data to capture the T${\;\;}_{\text{av}}$-range with approximately constant CV. Interestingly, CV exhibits a minimum in relation to T${\;\;}_{\text{av}}$ both in theoretical and experimental results.

We measured [Ca^2+^]_*i*_ spike trains in HEK293 stimulated with Carbachol (CCh) as described in Materials and methods. Stationary segments of spike trains as shown in Fig 2 were analysed with regard to ISI and amplitude sequences, from which we calculated averages and SDs.

We obtain a value of *α* from our experimental data by fitting it to the population data (Fig 6B). The plot of individual ISI SD- and T${\;\;}_{\text{av}}$ -data points of all cells reproduces the cumulant relation Eq 15. Fitting the linear function across the whole T${\;\;}_{\text{av}}$ range provides *α* = 0.231. Restricting the fit to ranges of small, intermediate and large values of T${\;\;}_{\text{av}}$ as specified in the caption of Fig 6B shows that we find a minimal *α* in the experimental data, which is consistent with our theoretical results (Fig 6A). We measured values of *α* in the range from 0.2 to 0.26 for HEK293 cells in an earlier study [9]. Here, we find values between 0.219 and 0.280, in agreement with the earlier results.

### The amplitude distribution

**Fig 7:**
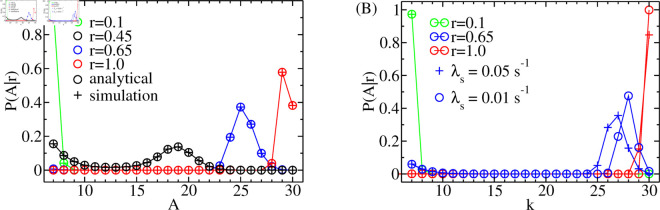
Amplitude distributions. We show the probability P(A|r) that A open clusters are reached during a spike given that $k_{sp}\;=\;7$ clusters are initially open, i.e. given the release event is a spike. A is a discrete variable; the lines are only a guide for the eye. (A) P(A|r) is affected by the degree of recovery *r* from negative feedback at the time of the occurrence of the spike. (B) The rate of growth of the negative feedback during the spike also affects P(A|r) (Eqs 5, 14). (A, B) Δ=0.95, *K*_*p*_ = 18, *s*_*p*_ = 2, *N*_*t*_ = 30, $i_{p}^{max}$ = 0.1, (A) *k*_*s*_ = 10, *g*_0_ = 1 s^−1^, $\lambda_{s}$ = 0.05 s^−1^, (B) *k*_*s*_ = 15, *g*_0_ = 0.6 s^−1^.

The spike amplitude is the maximum of open clusters reached during a spike. It is at least *k*_*sp*_ due to the definition of a spike. The distribution P(A|r) of amplitude A is affected by the degree of recovery *r* (Eq 6) at the spike time. We calculate P(A|r) from the probabilities that given a spike (initial state $k\;=\;k_{sp}$), the process reaches A$\;>\;k_{sp}$ before reaching 0. These splitting probabilities are determined by setting both 0 and A as absorbing states. Sect A of S1 Text explains the details. P(A|r) is the difference between the sequential splitting probabilities for reaching A. We verify this method of calculating the amplitude distributions by simulations in Fig 7A. Simulations and analytical calculations are indistinguishable.

If a spike occurs shortly after the previous one, the cell has only slightly recovered from the negative feedback that terminated the previous spike. The cluster open probability is small and the probability that the amplitude is larger than *k*_*sp*_ is negligible. Conversely, with almost complete recovery (r≈1), the cluster open probability is large and the probability of observing small amplitudes becomes insignificant. The dependency of P(A|r) on *r* connects P(A|r) to the ISI distribution *P*(*ISI*).

Once the spike has started, the negative feedback, which will terminate it, starts to grow with rate $\lambda_{s}$ (Eqs 5, 14). That rate has a moderate effect on the amplitude distribution (Fig 7B). The slower the negative feedback grows, the larger the spike amplitude. Since typical spike durations are in the range of 20 s, we use $\lambda_{s}$ = 0.05 s^−1^ in this study. In a future study, a detailed investigation of the role of $\lambda_{s}$ will be carried out in the context of spike modelling.

### The joint ISI-amplitude distribution P(A,ISI)

**Fig 8:**
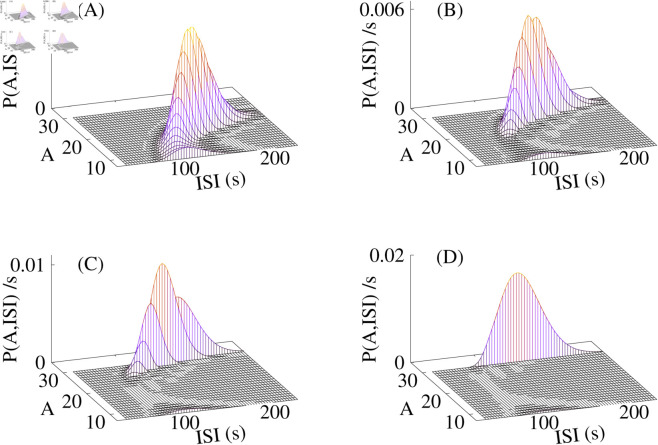
The joint ISI-amplitude distribution P(A,ISI) is the probability of observing a spike with amplitude A when the time ISI has passed since the onset of the previous spike. The number of open clusters *k*_*s*_ at which the CICR factor saturates has been increased from (A) to (D) to obtain distributions with different values of the correlation coefficient C_*c*_ (see Eq 12). Small amplitude spikes occur with some probability at small *k*_*s*_ early in the ISI (A). Essentially all-or-none spikes occur at large *k*_*s*_ values, only (D). Panel A shows a distribution typical for high correlation $C_{c}=\langle {\text{ISI}}_{i}|A_{i+1}\rangle$ between ISI_*i*_ and subsequent amplitude A_*i* + 1_. Panel D is typical of low correlation. (A) *k*_*s*_ = 6, C_*c*_ = 0.755, (B) *k*_*s*_ = 10, C_*c*_ = 0.589, (C) *k*_*s*_ = 15, C_*c*_ = 0.396, (D) $k_{s}\;>\;N_{t}$, C_*c*_ = 0.213, (A-D) Δ=0.95, *K*_*p*_ = 18, *s*_*p*_ = 2, *g*_0_ = 1 s^−1^, *N*_*t*_ = 30, $i_{p}^{max}$ = 0.1, *λ* = 0.01 s^−1^. Eq 10 has been used for CICR.

We calculate the probability of a spike with amplitude A${\;\;}_{i\;+\;1}$ following the i*th* ISI_*i*_ in this section. We can transform P(A|r) by $r=1\;-\;e^{-\lambda t_{sto}}$ and ISI = T${\;\;}_{\text{min}}$+*t*_*sto*_ into P(A|ISI). The joint probability is P(A,ISI) = P(A|ISI)P(ISI). We drop the indices of A and ISI in the distribution arguments for convenience of notation. Fig 8 shows examples of P(A,ISI). We calculated P(A|ISI) (see S1 Text, Eqs A.18, A.19 and A.21) and the moments of the ISI distribution (see S1 Text, Eq A.16 and A.17) analytically and then used Γ-distributions for P(ISI). We see distributions with high correlation between ISI_*i*_ and A_*i* + 1_ (Fig 8A) and weak correlation (Fig 8D). We get maximum-amplitude spikes across almost the whole ISI range in the case of weak correlation. Correlated spike trains show small-amplitude spikes at short ISI and large amplitudes at long ISI.

**Fig 9:**
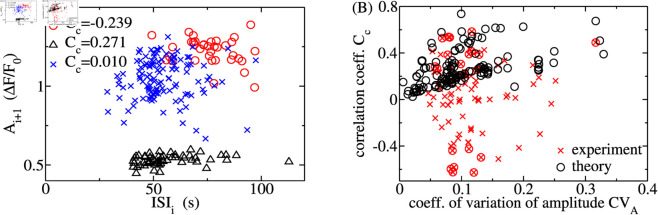
The relation of the i*th* ISI_*i*_ and the subsequent spike amplitude A_*i* + 1_. (A) A_*i* + 1_ versus ISI_*i*_ for typical spike trains with negative, positive, and vanishing Pearson correlation coefficient C_*c*_. All three of them exhibit weak or no correlation. (B) Pearson correlation coefficient C_*c*_ versus the coefficient of variation of the amplitude CV_*A*_ from theory with 136 parameter value sets and for all (36) measured spike trains with at least 15 ISIs. Each black and red data point represents a measured or simulated spike train, respectively. The C_*c*_ of encircled experimental data points has a p-value <0.05. Our theory provides only positive values of C_*c*_. The parameter values for the simulations and calculations are Δ=0.95, *g*_0_ = 40 s^−1^ *λ* = 0.005 s^−1^, all other parameter values have been varied, Eq 9 has been used.

We cannot directly verify the joint probability distribution with experimental data since that would require extremely long spike sequences. We therefore examine the correlation between ISI_*i*_ and subsequent amplitude A_*i* + 1_, instead. Fig 9A shows A_*i* + 1_ plotted against ISI_*i*_ for HEK293 spike trains with negative, positive, and vanishing correlation coefficients. These plots suggest a lack of a well-defined relationship between ISI_*i*_ and A_*i* + 1_.

Panel B of Fig 9 provides a more comprehensive analysis. It shows the Pearson correlation coefficients $C_{c}=\langle {\text{ISI}}_{i}|A_{i+1}\rangle$ and the coefficient of variation of the amplitude CV_*A*_ for spike trains measured with more than 15 spikes and theoretical results. The measured spike trains exhibit positive and negative *C*_*c*_ with absolute values ranging from 0.034 to 0.615. The p-values of most of the correlation coefficients are larger than 0.05. We encircled those with p-values smaller than 0.05, among which we find values close to 0.6, -0.6, and 0.22, i.e. correlation between ISI_*i*_ and A_*i* + 1_, anticorrelation, and lack of correlation. Fig 9B shows that theory reproduces the range of positive values.

Our theory formulates the idea that the recovery from the negative feedback that terminated the previous spike increases the IP_3_R open probability or spike amplitude with increasing ISI. Consequently, it produces only positive *C*_*c*_ for ISI_*i*_ and A_*i* + 1_. We find both correlation (C${\;\;}_{c}\approx$0.6) and lack of correlation (C${\;\;}_{c}\;<\;$0.25) in our calculations and experiments. Hence, contrary to our expectations, recovery from negative feedback does not necessarily cause a correlation between ISI_*i*_ and A_*i* + 1_.

There are experimental spike trains that exhibit vanishing C_*c*_ in a wide range of CV_*A*_ and T${\;\;}_{\text{av}}$. Theoretical C_*c*_s vanish only at small values of CV_*A*_ or at very large T${\;\;}_{\text{av}}$ (T${\;\;}_{\text{av}}$$\;\gg \;\lambda^{-1}$, see the black circle at CV≈0.2 and C_*c*_≈0 in Fig 9B). This observation, together with the negative correlation coefficients found in our experiments, is a strong indication of processes affecting the correlation between ISIs and amplitudes, which are not included in our theory, yet.

### Remarks on quantifying parameter values in the face of large cell variability

IP_3_-induced Ca^2+^ spiking shows characteristics that are not subject to cell variability, such as *α* and the agonist sensitivity *γ* of the concentration-response relation of T${\;\;}_{\text{av}}$ [9], and spike train properties like T${\;\;}_{\text{av}}$, the ISI SD *σ*, amplitudes and *C*_*c*_ with large cell variability. This entails a corresponding set of parameter values that most affect variable properties (*N*_*t*_, *s*_*p*_, *k*_*s*_, *K*_*p*_) and thus quantify cell variability and another set of parameters relating to ’conserved’ values. The latter are *λ* and *g*_0_ in our theory, due to the properties of *α*. Note that the value of *γ* is not restricted by the current level of theory and therefore can always be met.

The purpose of theory is to reproduce both the values of the conserved properties and the value ranges of the variable properties, and thus to provide a quantitatively consistent formulation of a mechanistic hypothesis of spike generation including both. Theory should also be able to reproduce results for a specific set of variable properties of a given cell by specifying the values of parameters that describe cell variability. Table 1 shows three examples that we can also fit individual cells. Those example cells have been chosen to have more than 15 spikes, positive C_*c*_, and to represent short, intermediate and long T${\;\;}_{\text{av}}$. We specify all parameter values for the three cells in the table. We used the value of *α*, its robustness properties, and the T${\;\;}_{\text{av}}$ range to specify *λ*, *g*_0_ and the range of *K*_*p*_. We used T${\;\;}_{\text{av}}$- and *C*_*c*_-values to quantify the parameters describing cell variability. It was easier to fit the robustness properties of *α* and the properties of the specific cells in Table 1 when we used Eq 11 instead of Eq 10.

**Table 1:** Fits for three example spike trains from Fig 2. Parameter values: Fig 2A, segment 1: $K_{p}$ = 23, $k_{s}$ = 10, $s_{p}$ = 2.75, $i_{p}^{max}$ = 1.0; Fig 2B, segment 2: $K_{p}$ = 22, $k_{s}$ = 13, $s_{p}$ = 1.19, $i_{p}^{max}$ = 0.73; Fig 2C: $K_{p}$ = 18, $k_{s}$ = 14, $s_{p}$ = 0.61, $i_{p}^{max}$ = 0.118. The parameter values common to all three cells are $N_{t}$ = 30, Δ = 0.95, $g_{0}$ = 40 s^−1^ λ = 0.005 s^−1^. Eq 11 has been used for CICR. The units of T${\;\;}_{\text{av}}$ and T${\;\;}_{\text{min}}$ are seconds. The values for T${\;\;}_{\text{min}}$ are estimates from the experimental records. The theoretical T${\;\;}_{\text{min}}$ -values are parameters and not results of calculations. Experimental values of α of individual cells cannot be determined since we do not know individual T${\;\;}_{\text{min}}$-values. N${\;\;}_{sp}$ is the number of spikes. Theoretical results are moments of distributions which do not have N${\;\;}_{sp}$ as parameter (n.a.).

	Fig 2A, segment 1		Fig 2B, segment 2		Fig 2C	
	exp.	theo.	exp.	theo.	exp.	theo.
T${\;\;}_{\text{av}}$	56.7	56.5	97.9	97.6	238.9	236.4
C_*c*_	0.271	0.276	0.215	0.206	0.296	0.287
CV_*A*_	0.060	0.049	0.093	0.096	0.129	0.143
*α*		0.222		0.193		0.185
N${\;\;}_{\text{sp}}$	57	n.a.	19	n.a.	20	n.a.
T${\;\;}_{\text{min}}$	15	15	20	20	20	20

## Discussion

We present a stochastic model of IP_3_-induced Ca^2+^ spiking in HEK293 cells and parameterise it through experiments. The model takes into account the slow deterministic behaviour of globally averaged feedback variables. Transition probabilities depend non-linearly on the feedback variables. Analytical theory required to find a method to solve the corresponding time-dependent Master Equation. We generalised a linear method [66] to a polynomial dependency by reducing a system of higher-order difference equations to a system of first-order difference equations. The generalisation now allows for a very broad spectrum of dependencies of transition probabilities on the state of the system and dynamic feedback variables.

We calculate the first and second moments of the ISI distribution analytically. Since simulated ISI-distributions with *α*-values applying to HEK293 cells are very well approximated by Γ distributions in agreement with experimental results [14], we can completely determine ISI statistics analytically. The same applies to the amplitude statistics. That puts us in the position to calculate ISI-amplitude correlations analytically, which we can also determine from measured spike sequences. Hence, our approach substantially expands the possibilities to relate analytical calculations to experimental results.

We derived transition rate expressions for CICR and sensitisation of IP_3_Rs by [IP_3_]. The dependency of the puff rate on [IP_3_] has been measured by Dickinson et al. [35] and we used their results (Eq 3). The CICR factor has been derived as the inverse of the first passage time from the bare receptor to the state with either 3 or 4 Ca^2+^ ions bound (Eqs 10, 11). Furthermore, we can now simultaneously include several time-dependent feedback. Each feedback may have its own relaxation rate, as long as the ratio of rates is a rational number.

That implicates progress toward a theory closer to the experiment. The parameter values, which we obtain as part of the results of the theory, now correspond more to expectations based on average ISIs. For example, the rate of recovery from negative feedback *λ* sets the slope *α* of the cumulant relation Eq 15 [67,90]. The fit of earlier experimental data with our previous models resulted in *λ* = 1.44 s^−3^ [67]. Such a recovery time of about 700 s was required to meet the robustness properties, but it appears rather long compared to average ISIs. In those earlier models, we used a simple power function dependency of the CICR factor on the number of open clusters. With time-dependent [Ca^2+^]_*i*_ and our current description of CICR by Eqs 10 and 11, the new parameter *K*_*p*_ is part of the description of CICR. It also affects *α*. This additional degree of freedom in fixing *α* allows more realistic values of *λ* (Fig 6A). The measured *α*-values in this study agree very well with earlier measurements [9]. That confirms our conclusion that the slope of the cumulant relation is a characteristic feature of spike trains elicited via a specific GPCR in a given cell type, that *α* is robust against many perturbations and is not subject to cell variability.

We found better agreement of *α* robustness properties and correlation coefficients with the expression of the CICR factor assuming binding of 4 Ca^2+^ ions to reach high open probability (Eq 11) than with binding of 3 ions. Both expressions, Eq 10 and Eq 11, are essentially linear for large [Ca^2+^]. Interpretation of this result needs to take into account that CICR may include Ca^2+^ dependencies in addition to the mass action kinetics we included here as, for example, the IP_3_R modelling study by Siekmann et al. illustrates [72]. The assumption that 4 ions need to bind entails a very small transition probability at small [Ca^2+^], which then rises quickly with increasing [Ca^2+^]. It thus entails a sharper threshold of spike initiation compared to the binding of 3 ions. Our results suggest highly non-linear rate expressions with sharp thresholds to correspond to experimental observations. However, this highly non-linear behaviour of cells might not be due to mass action kinetics alone.

The experimental and theoretical results for the Pearson correlation coefficient C_*c*_ are, to some degree, unexpected. Expectations are shaped by the ideas on feedback during a spike sequence, which defines our theory. Upon onset of a spike, negative feedback is initiated, e.g. by ER depletion or diminution of IP_3_ production, and finally terminates the spike. Afterwards, the cell recovers from it, which causes a continuous increase of the spike probability up to some saturation value. This suggests a correlation between an ISI and the amplitude of the subsequent spike. Some spike trains exhibit such a correlation ($C_{c}\geq 0.4$). However, many more show weak correlation ($0.25\leq C_{c}\leq 0.4$), no correlation ($|C_{c}|\leq 0.25$), or even anticorrelation ($C_{c}\leq -0.25$). These correlation properties are subject to cell variability, i.e. HEK293 cells stimulated identically can exhibit any of the four cases. This suggests that correlation is not only a characteristic of feedback triggered by muscarinic receptor activation, but is also strongly affected by cell-specific properties, which are subject to cell variability.

A negative ISI-amplitude correlation could be explained by decreasing IP_3_R open probability at large ISI due to a very slowly decaying positive feedback component during the spike. However, that would substantially increase *α* in Eq 15 away from measured values [88]. The challenge, therefore, is to find feedback mechanisms providing both $C_{c}\;<\;0$ and α≪1. The analytical stochastic theory presented in this study, will be of value for testing hypotheses on the pathway components that cause this anticorrelation since it can include a large variety of positive and negative feedback mechanisms.

In general, mathematical theory is a formulation of our mechanistic and quantitative hypotheses on the system under consideration. Here, we parametrise the model using both general statistical properties and robustness properties. In addition, we specified it to spike trains from three representative cells. However, the resulting parameter sets for the specific spike trains in Table 1 may not be unique. Identifiability in such systems requires at least as many independent single-cell experiments as there are parameters to estimate, each yielding sufficiently long spike trains for robust moment statistics. This is not practically achievable, as the cell state does not remain constant over such a long time (see Fig 2), thereby limiting the ability to formulate a complete hypothesis.

These constraints raise a broader question: What can be inferred about a stochastic, highly variable biological system, and what level of resolution is necessary for functional understanding? While a complete quantitative theory tailored to individual cells could offer detailed insights, its utility is constrained by the high degree of cell variability. In contrast, the robustness of function across heterogeneous cells suggests that essential system behaviours are governed by invariant properties rather than cell-specific parameters.

Theoretical frameworks that incorporate stochasticity and biological variability - while remaining faithful to function and the general statistical features of the system - may not be fully predictive at the single-cell level. However, they can still elucidate the core design principles that ensure functional robustness. Such an approach may help distinguish biological systems from engineered technological systems, the former being characterised by resilience to parameter variability, the latter requiring precise composition. In the long term, incomplete yet principled models of this nature may provide critical insights into the architectural logic of living systems.

## Materials and methods

### Materials

Dulbecco’s Modified Eagle’s Medium (DMEM) supplemented with 4.5 g/L D-glucose and L-glutamine, and FluoroBrite DMEM were from Thermo Fisher Scientific. Calbryte™ 520 AM was purchased from AAT Bioquest, and carbamoylcholine (carbachol; CCh) was sourced from Merck (Sigma-Aldrich). 35 mm glass-bottom dishes for imaging were from ibidi GmbH.

### Single-cell imaging of [Ca^2+^]_*i*_ in HEK293 cells

HEK293 cells were cultured in DMEM supplemented with 10% fetal bovine serum (FBS). For imaging experiments, the cells were plated onto 35-mm glass-bottom petri dishes; loaded with CalBryte™ 520 AM (2.5 μM) for 30 minutes at 37 °C, washed with fresh DMEM, and incubated again for an additional 30 minutes before imaging to allow for de-esterification of the indicator. Single-cell fluorescence measurements were performed at 30 °C under humidity and 5% CO_2_ control in conditioned FluoroBrite DMEM supplemented with 10% FBS and 4 mM L-glutamine to better preserve cell viability during the extended acquisition. CCh was added to the cells by pipette 15 minutes after the recording started at varying concentrations (3 μM, 5 μM, 10 μM, and 15 μM) across different experiments. Cells were imaged using a Nikon inverted microscope equipped with a spinning disk unit (ANDOR CSU-W1) featuring a 50 μm fixed-size pinhole. A Plan Fluor 20x Mimm DIC N2 objective (NA 0.75) was used for image acquisition. Detection was carried out using an Andor iXon DU-888 EMCCD camera (1024x1024 pixels) with a 525/45 nm emission filter, an EM gain of 300, no binning, a 30 ms exposure time, and readout mode (EM Gain) set to 30 MHz at 16-bit. Excitation was achieved using a 488 nm solid-state laser at 5% of its maximum power (0.1 mW at the objective). This setup allowed for a sufficient contrast while limiting phototoxicity. Images were acquired at 1-second intervals. Recordings lasted between 105 and 150 minutes to maximise Ca^2+^ spike detection per cell. Examples of recorded spike trains are shown in Fig 2.

### Analysis of [Ca^2+^]_*i*_ spike trains

Time-lapse image series were analysed with Nikon’s NIS-Elements AR software (version 5.21.03) [91], which was used to define regions of interest (ROIs) and extract fluorescence intensity measurements. ROIs were drawn following the contours of the cells and their shape was adjusted over time using the ’Edit ROIs in Time’ tool to match cell rearrangements during acquisition. We performed baseline correction of the spike trains using the PeakUtils Python package [92] and then normalised the intensities to the average corrected baseline (F_0_). Ca^2+^ spikes were detected and characterised using the PyCaSig software [93]. This GUI-based Python program automates the processing of [Ca^2+^]_*i*_ time series data and computes measures of spike properties. As previously described [9], only the stationary components of spike sequences were considered for ISI analysis.

In our experiments, HEK293 cells are subjected to prolonged CCh stimulation, which can cause receptor desensitisation and other slow processes that lead to changes in the average ISIs throughout the duration of a spike train [94,95]. Slow trends on the time scale of a few times the average ISI entail contributions, especially to higher moments and cumulants, which are not related to the stochastic aspects of spike generation. We applied two measures to avoid these contributions: if a spike train exhibits segments comprising several ISIs with different average ISIs, we analysed those segments separately as further explained in S1 Text, Sect D. Such segments are marked in Fig 2A, 2B and 2D. Furthermore, residual linear trends within these segments were removed before the calculation of the standard deviation and correlation coefficients. The removal of trends did not change the average (S1 Text, Sect D). Only stationary sequences of spike trains with at least 11 recorded spikes were included in the analysis of average ISI and with at least 15 spikes in the analysis of SD and correlations.

## Supporting information

S1 Text Section A. Calculating moments of the first passage time distribution. Section B. Numerical methods. Section C. Comments on the CICR-factor *r*_*n*_. Section D. Comments on determining stationary ISI sequences.
(PDF)
