# Supplementary material for: Stochastic model of IP3-induced Ca2+ spiking of HEK293 cells
Source: PLoS Comput Biol. 2025 Aug 22;21(8):e1013322. doi: 10.1371/journal.pcbi.1013322 (PMC12373179; doi:10.1371/journal.pcbi.1013322)
Supplement: S1 Text — Section A. Calculating moments of the first passage time distribution. Section B. Numerical methods. Section C. Comments on the CICR-factor rn. Section D. Comments on determining stationary ISI sequences. (PDF) [file pcbi.1013322.s001.pdf]

Supporting information

July 28, 2025

A Calculating moments of the first passage time distribution

An analytic solution for the moments of the first passage time distribution with N_p=1 in the form of a sum of matrix products has been worked out in Falcke and Friedhoff [1]. We show here that the case $N_p > 1$ can be also transformed into a system of first order difference equations for which this solution holds (Fig 2, main text).

We start with the Master Equation for the state probabilities P_k

$$\frac{dP_0(t)}{dt} = \delta P_1(t) - \Psi_{0,1}(t)P_0(t), \tag{A.1}$$

$$\frac{dP_k(t)}{dt} = \Psi_{k-1,k}(t)P_{k-1}(t) + (k+1)\delta P_{k+1}(t) - \left(\Psi_{k,k+1}(t) + k\delta\right) P_k(t), \tag{A.2}$$

with $k = 1, \ldots, N_t - 1$. The $\Psi_{k,k+1}$ are polynomials in $e^{-\lambda t}$

$$\Psi_{k,k+1} = \sum_{j=0}^{N_p} \psi_{k,k+1}^{(j)} e^{-j\lambda t}. \tag{A.3}$$

The number of states to be included in the Master Equation depends on the specific problem considered (first passage, splitting probability). We denote this number by M.

To reach a matrix notation we define coefficient matrices

$$D^{(0)}_{k+1,k} = \psi^{(0)}_{k-1,k}, \tag{A.4}$$

$$D^{(0)}_{k,k+1} = k\delta, \tag{A.5}$$

$$D^{(0)}_{k,k} = -\left(\psi^{(0)}_{k-1,k} + (k-1)\delta\right), \tag{A.6}$$

$$D^{(j)}_{k+1,k} = -\psi^{(j)}_{k-1,k}, \tag{A.7}$$

$$D^{(j)}_{k,k} = \psi^{(j)}_{k-1,k}, \tag{A.8}$$

and all other matrix elements vanish. Note that the index of the matrix elements starts with 1, and the index of the transition probabilities with 0.

The initial state i_s defines the vector r with $r_{i_s} = 1,\, r_k = 0,\ k \neq i_s$. The Laplace transform of the Master equation allows for a comfortable calculation of moments of the first-passage times. The Laplace transform of Eq (A.1) is the system of linear difference equations [1]

$$s\tilde{P}_k(s) - r_k = \sum_{m=1}^{M} D^{(0)}_{k,m}\tilde{P}_m(s) + \sum_{j=1}^{N_p}\sum_{m=1}^{M} D^{(j)}_{k,m}\tilde{P}_{(j-1)*M+m}(s+\lambda). \tag{A.9}$$

$$\tilde{P}(s)_{j*M+k}(s) = \tilde{P}(s)_{(j-1)*M+k}(s+\lambda), \quad j = 1, \ldots, N_p - 1. \tag{A.10}$$

We define the matrices

$$E(s) = \begin{bmatrix} \mathbb{1}s - D^{(0)} & \mathbb{0} \\ \mathbb{0} & \mathbb{1} \end{bmatrix} \tag{A.11}$$

$$D = \begin{bmatrix} D^{(1)} & \ldots & D^{(N_p)} \\ \mathbb{1} & & \mathbb{0} \end{bmatrix} \tag{A.12}$$

$$B(s) = E(s)^{-1}D \tag{A.13}$$

with dimension $MN_p \times MN_p$, and obtain [1]

$$\tilde{P}(s) = E^{-1}r + \sum_{k=1}^{\infty}\prod_{j=0}^{k-1} B(s+j\lambda)E(s+k\lambda)^{-1}r \tag{A.14}$$

as the solution of Eqs (A.9), (A.10). Note that if we also allowed the left-going

transitions in the state chain (Fig 2, main text) to be polynomials in $e^{-\lambda t}$, only the definitions of E and D would change. However, the solution Eq A.14 would still apply. The same applies to more general state schemes than a linear one.

If we consider the first passage from state 0 to N_t, the vector r is $r_0 = 1$, $r_k = 0,\ k \neq 0$, and we set $P_{N_t}(t) = 0$ or equivalently $\Psi_{N_t,N_t-1} = 0$. N_t is then an absorbing state, i.e., the transition probability out of this state is 0. The first passage time (t_f) distribution $F_{0,N_t}(t_f)$ is given by the probability flux out of the state range from 0 to $N_t - 1$:

$$F_{0,N_t}(t_f) \quad = \quad -\frac{d}{dt}\sum_{k=0}^{N_t-1} P_k(t)\Bigg|_{t=t_f} \quad = \quad \Psi_{N_t-1,N_t} P_{N_t-1}. \tag{A.15}$$

The Laplace transform of the first-passage-time distribution is [1]

$$\tilde{F}_{0,N_t}(s) \quad = \quad \sum_{j=0}^{N_p} \psi^{(j)}_{N_t-1,N_t} \tilde{P}_{N_t-1}(s + j\lambda), \tag{A.16}$$

The moments of the first-passage-time distribution are given by [2]

$$\langle {t_f}^i \rangle = (-1)^i \frac{\partial^i}{\partial s^i} \tilde{F}_{0,N_t}(s)\Bigg|_{s=0}. \tag{A.17}$$

We calculate the moments of the ISI distribution as the moments of the FPT distribution from state 0 to state k_{sp}. The equations apply for this calculation with N_t replaced by k_{sp}. We use Eqs 4 and 13 applying to the recovery phase to determine $\psi^{(j)}_{k,k+1}$ for ISI calculations.

The distribution P_A of the amplitude A is calculated from splitting probabilities. The initial state i_s is the minimum number of open clusters to be considered a spike k_{sp}. State 0 is an absorbing state. Transition probabilities out of absorbing states are 0, that is, $P_0(t) = 0$ or equivalently $\psi^{(j)}_{0,1} = 0$, $j = 0, \ldots N_p$. We consider all states a, $i_s < a \leq N_t$ in sequential calculations also as absorbing states, that is we set $P_a(t) = 0$ or equivalently $\Psi_{a,a-1} = 0$. The matrices $E(s)$ and D for this splitting probability problem also obey Eqs A.4-A.8, A.11, A.12 with the transition probabilities out of the absorbing state 0. We use Eqs 5 and 14 applying during the spike to determine the $\psi^{(j)}_{k,k+1}$ for amplitude calculations. The solution of the splitting probability problem is

then given by Eq A.14. The Laplace transformation of the distribution of the time till absorption in either 0 or a is

$$\tilde{F}_{i_s,0,a}(s) \quad = \quad \delta\tilde{P}_1(s) + \sum_{j=0}^{N_p} \psi_{a-1,a}^{(j)} \tilde{P}_{a-1}(s+j\lambda). \tag{A.18}$$

The probability to be absorbed by state a is

$$\tilde{F}_{i_s,a}(0) \quad = \quad \sum_{j=0}^{N_p} \psi_{a-1,a}^{(j)} \tilde{P}_{a-1}(j\lambda), \quad i_s < a \leq N_t. \tag{A.19}$$

and by state 0 is

$$\tilde{F}_{i_s,0}(0) \quad = \quad \delta\tilde{P}_1(0). \tag{A.20}$$

$\tilde{F}_{i_s,a}(0)$, which is also the probability that the amplitude A is larger than $a-1$. The probability P_A that the amplitude is equal to A is

$$P_A \quad = \quad \tilde{F}_{i_s,A}(0) - \tilde{F}_{i_s,A+1}(0), \quad \tilde{F}_{i_s,N_t+1}(0) \equiv 0. \tag{A.21}$$

B Numerical methods

B.1 Evaluation of Eq A.14

If N is large and the ratio between the fastest state transition rate and the relaxation rate λ is large, high precision of the numerical calculations is required for the use of Eq (A.14). *A priori* known values like $\tilde{F}_{0,N_t}(s=0) = 1$ can be used to monitor the convergence of the calculations. The matrix products become very large at intermediate values of $j\lambda$ during the summation in Eq (A.14) and their sign alternates such that two consecutive summands nearly cancel. Intermediate summands are of order larger than 10^{17} and thus we face a loss of significant figures even with the numerical floating-point number format long double. We used Arb, a C numerical library for arbitrary-precision interval arithmetic [3], to circumvent this problem. It allows for arbitrary precision in calculations with Eq (A.14). The software is available in the supplemental material of Friedhoff et al. [4]. Computational speed is the only limitation with this library and has

determined the parameter range for which we established analytical results. We were able to go to a time-scale separation of $\approx 10^{-6}$ using this library. We calculated moments with a relative precision of $4 \cdot 10^{-4}$. Arbitrary precision computations with Arb take between 1 s and 10 days, heavily dependent on the matrix dimensions set by the index of the absorbing state. See Falcke and Friedhoff [1] for more detail.

B.2 Simulation method

We also simulated the state chain stochastically using a Gillespie algorithm in C++ to verify our analytical results. We used the algorithm for time-dependent propensities by Alfonsi et al. [5]. Typically 10^5 trajectories were simulated to determine distributions for one parameter set.

C Comments on the CICR-factor r_n

The large range of Hill coefficients measured in experiments (cited in the main text) does not allow a definite statement on the number of activating Ca^{2+}-binding sites that need to be occupied to reach a channel state with high open probability. Recent research has shown that IP_3R tetramere has (at least) 4 activating Ca^{2+}binding sites but also describes this stoichiometric question as not resolved yet [6]. Given the situation, we consider it to be the most reasonable to start from simple mechanistic ideas when deriving rate expressions for Ca^{2+} -dependent activation of IP_3R. We assume that activating Ca^{2+}-binding is faster than inhibitory Ca^{2+}-binding and that the activating binding site has a higher affinity than the inhibitory one. Therefore, inhibition can be neglected at low [Ca^{2+}]. We also assume identical Ca^{2+}-binding sites. Note that we do not require cooperativity.

We call the probability that i Ca^{2+}-binding sites are occupied $P_b(i)$ as in the main

text. Its Master Equation is

$$
\begin{aligned}
\frac{dP_b(0)}{dt} &= k^- P_b(1) - 4\bar{k}^+ P_b(0) \\
\frac{dP_b(1)}{dt} &= 4\bar{k}^+ P_b(0) + 2k^- P_b(2) - (k^- + 3\bar{k}^+)P_b(1) \\
\frac{dP_b(2)}{dt} &= 3\bar{k}^+ P_b(1) + 3k^- P_b(3) - (2k^- + 2\bar{k}^+)P_b(2) \\
\frac{dP_b(3)}{dt} &= 2\bar{k}^+ P_b(2) + 4k^- P_b(4) - (3k^- + \bar{k}^+)P_b(3) \\
\frac{dP_b(4)}{dt} &= \bar{k}^+ P_b(3) - 4k^- P_b(4)
\end{aligned}
$$

The stationary states $P_b^0(i)$ are

$$
\begin{aligned}
M(c) &= c^4 + 4K_p c^3 + 6K_p^2 c^2 + 4K_p^3 c + K_p^4 \\
P_b^0(0) &= \frac{K_p^4}{M(c)} \\
P_b^0(1) &= \frac{4K_p^3 c}{M(c)} \\
P_b^0(2) &= \frac{6K_p c^2}{M(c)} \\
P_b^0(3) &= \frac{4K_p c^3}{M(c)} \qquad \text{(C.1)} \\
P_b^0(4) &= \frac{c^4}{M(c)} \qquad \text{(C.2)}
\end{aligned}
$$

K_p has been defined in the context of Eq. 6 in the main text. If IP_3R needs to bind at least 3 Ca^{2+} ions to reach a channel state with high open probability, the stationary open probability is proportional to $P_b^0(3) + P_b^0(4)$.

The relation of the open probability to [Ca^{2+}] measured in experiments has often been fit to Hill equations $c^{n_h}/(K_h^{n_h} + c^{n_h})$ in the range of small c. We illustrate in Fig. C.1 that such fits may result in n_h values smaller than the number of binding sites occupied at high open probability.

We provide some intermediate steps of the calculation that lead to the expression for

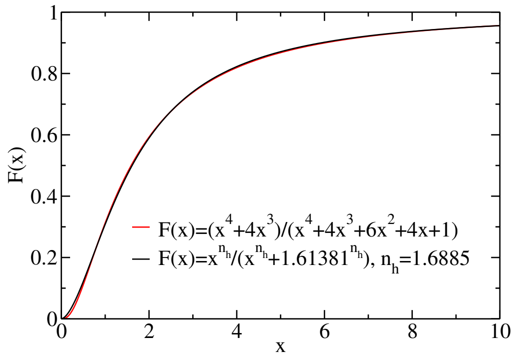

Fig C.1. Fitting $P_b^0(3) + P_b^0(4)$ (see Eqs C.1, C.2) by a Hill function $x^{n_h} / \left(\left(\frac{K_h}{K_p} \right)^{n_h} + x^{n_h} \right)$, with $x = \frac{c}{K_p}$. We obtain a good fit by n_h=1.6885 and $K_h = 1.61318 K_p$. The fit has been done with the fitting tool of the plotting software xmgrace.

r_3. The Laplace transform with the Laplace variable s of the first passage problem is

$$\begin{aligned} (s + 4\bar{k}^+)\tilde{P}_b(0) - k^- \tilde{P}_b(1) &= 1 \\ (s + k^- + 3\bar{k}^+)\tilde{P}_b(1) - 4\bar{k}^+ \tilde{P}_b(0) - 2k^- \tilde{P}_b(2) &= 0 \\ (s + 2(k^- + \bar{k}^+))\tilde{P}_b(2) - 3\bar{k}^+ \tilde{P}_b(1) &= 0. \end{aligned}$$

Solving for $\tilde{P}_b(2)$ results in

$$2\bar{k}^+ \tilde{P}_b(2) = \frac{24\bar{k}^{+3}}{(s + 4\bar{k}^+)\left[(s + k^- + 3\bar{k}^+)(s + 2(k^- + \bar{k}^+)) - 6k^-\bar{k}^+\right] - 4k^-\bar{k}^+(s + 2(k^- + \bar{k}^+))}.$$

The rate r_3 is then

$$r_3 = -\left[2\bar{k}^+ \left. \frac{d\tilde{P}_b(2)}{ds} \right|_{s=0} \right]^{-1}. \tag{C.3}$$

D Comments on determining stationary ISI sequences

An important aspect to take into account for spike analysis, evident by looking at traces, is how, during prolonged constant stimulation, the spiking frequency of cells is not always stable: typically, a higher spike frequency is observed at the beginning of the recording, followed by a reduced rate over time - it has to be considered that over hours, prolonged exposure to stimulant can induce subtype-specific effects on the regulation of

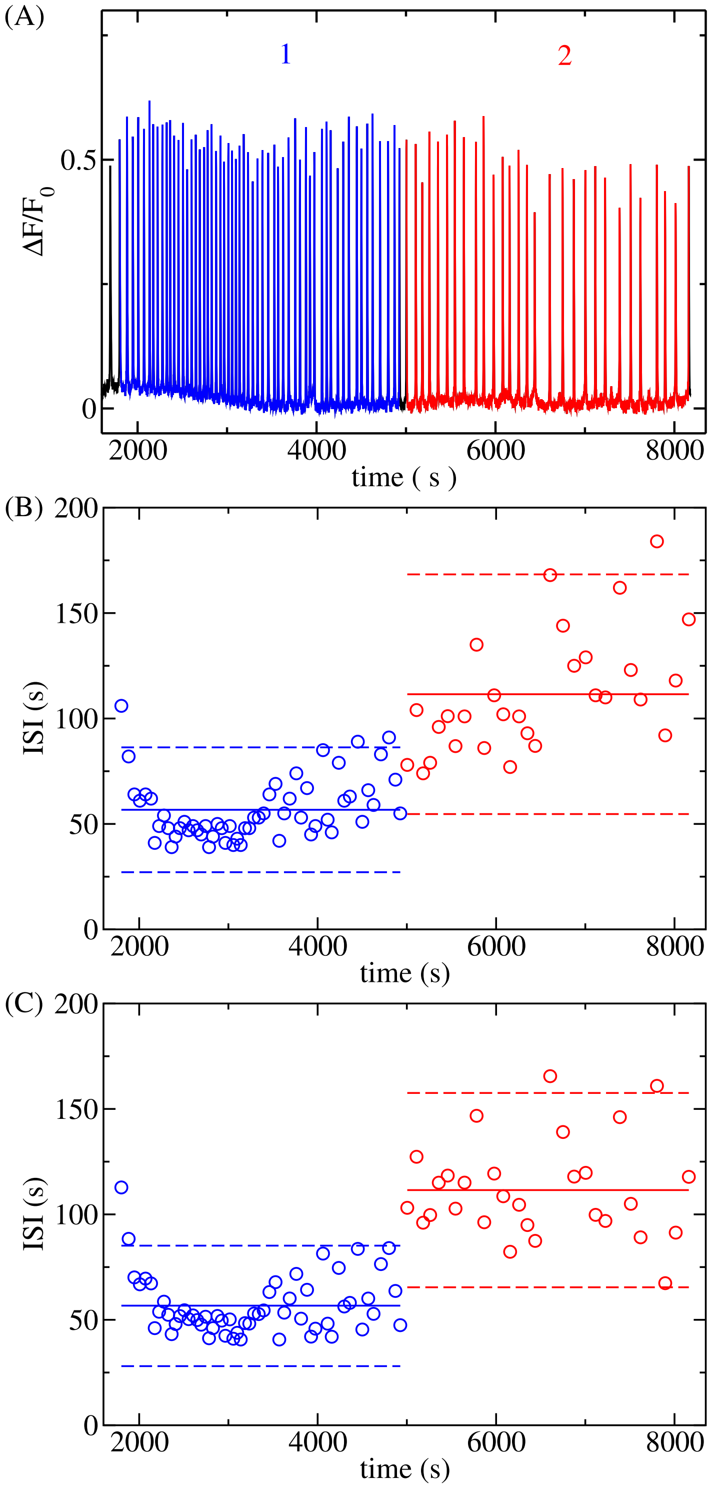

Fig D.1. (A) Panel A of Fig. 2 replotted. (B) ISI values (∘) of the sequences of ISI selected in A. (C) The ISI sequences after removing a linear trend to reduce contributions to the standard deviation not caused by the stochastic process. In (B) and (C), blue corresponds to sequence 1, red to sequence 2, full line is average ISI, dashed lines are average ± 2·SD.

muscarinic receptors [7, 8], causing changes in average ISIs. As previously described [9], our analysis of moments of ISI sequences is meaningful only for stationary sequences; therefore, when a spike train clearly displays more than one frequency, we do not analyse it in its entirety, but select stationary sequences.

The identification of stationary ISI sequences uses their averages and standard deviations (SDs) to distinguish them. It was guided by the criteria:

1. The average ISI of a given stationary sequence must be clearly different from neighbouring sequences. We required that it is outside the interval average ISI ± 2·SD of neighboring ISI sequences (see Fig. D.1),

2. ISI sequences should be as long as possible.

However, these criteria can narrow the range of the boundary location between sequences down to a few ISIs, but cannot identify the exact location of the sequence boundary down to a single ISI. The first criterion involves the SD converging like $N_I^{-\frac{1}{2}}$ with the number of ISIs in a sequence $N_I^{-\frac{1}{2}}$. Identification of a specific ISI (to be added) as the last or first one in a sequence requires a precision of N_I^1. Hence, the required precision increases faster than the SD converges and we cannot expect a stable algorithm from the criterion.

Therefore, we selected the final location of the boundary by visual inspection. In most cases, we considered the range of ISI values, where the boundary could be placed according to criterion 1 and chose the middle. Some cells required time to adjust to a new rate; as a consequence, the distinction between intervals was ambiguous over several ISIs, and we omitted some of them to account for this transitory state. In some other cases, spikes were included in a spike frequency group even when alternatives could have been justified; here, we considered the surrounding ISI values and placed the interval boundary at the point where the ISIs began to cross the calculated threshold.

References

1. Falcke M, Friedhoff VN. The stretch to stray on time: Resonant length of random walks in a transient. Chaos: An Interdisciplinary Journal of Nonlinear Science. 2018;28(5):053117.

2. van Kampen NG. Stochastic Processes in Physics and Chemistry. Amsterdam: North-Holland; 2001.

3. Johansson F. Arb: efficient arbitrary-precision midpoint-radius interval arithmetic. IEEE Transactions on Computers. 2017;66:1281-92.

4. Friedhoff VN, Lindner B, Falcke M. Modeling IP_3-induced Ca^{2+} signaling based on its interspike interval statistics. Biophysical Journal. 2023;122(13):2818-31.

5. Alfonsi A, Cancès E, Turinici G, Ventura BD, Huisinga W. Exact simulation of hybrid stochastic and deterministic models for biochemical systems. INRIA Rapport de Recherche, Thèmes NUM et BIO. 2004;5435.

6. Arige V, Terry LE, Wagner LE, Malik S, Baker MR, Fan G, et al. Functional determination of calcium-binding sites required for the activation of inositol 1,4,5-trisphosphate receptors. Proceedings of the National Academy of Sciences. 2022;119(39):e2209267119. Available from: https://www.pnas.org/doi/abs/10.1073/pnas.2209267119.

7. Mundell SJ, Benovic JL. Selective regulation of endogenous G protein-coupled receptors by arrestins in HEK293 cells. Journal of Biological Chemistry. 2000 Apr;275(17):12900-8.

8. Stope MB, Kunkel C, Kories C, Schmidt M, Michel MC. Differential agonist-induced regulation of human M2 and M3 muscarinic receptors. Biochemical Pharmacology. 2003 Dec;66(11):2099-105.

9. Thurley K, Tovey SC, Moenke G, Prince VL, Meena A, Thomas AP, et al. Reliable Encoding of Stimulus Intensities Within Random Sequences of Intracellular Ca^{2+} Spikes. Sci Signal. 2014;7(331):ra59.
